# Promoting Artificial Intelligence for Global Breast Cancer Risk Prediction and Screening in Adult Women: A Scoping Review

**DOI:** 10.3390/jcm13092525

**Published:** 2024-04-25

**Authors:** Lea Sacca, Diana Lobaina, Sara Burgoa, Kathryn Lotharius, Elijah Moothedan, Nathan Gilmore, Justin Xie, Ryan Mohler, Gabriel Scharf, Michelle Knecht, Panagiota Kitsantas

**Affiliations:** Charles E. Schmidt College of Medicine, Florida Atlantic University, Boca Raton, FL 33431, USA; dlobaina2021@health.fau.edu (D.L.); sburgoa2022@health.fau.edu (S.B.); klotharius2022@health.fau.edu (K.L.); emoothedan2022@health.fau.edu (E.M.); ngilmore2022@health.fau.edu (N.G.); jxie2018@health.fau.edu (J.X.); rmohler2022@health.fau.edu (R.M.); gscharf2016@health.fau.edu (G.S.); kebam@health.fau.edu (M.K.); pkitsanta@health.fau.edu (P.K.)

**Keywords:** artificial intelligence, machine learning, breast cancer screening, risk prediction, women

## Abstract

**Background:** Artificial intelligence (AI) algorithms can be applied in breast cancer risk prediction and prevention by using patient history, scans, imaging information, and analysis of specific genes for cancer classification to reduce overdiagnosis and overtreatment. This scoping review aimed to identify the barriers encountered in applying innovative AI techniques and models in developing breast cancer risk prediction scores and promoting screening behaviors among adult females. Findings may inform and guide future global recommendations for AI application in breast cancer prevention and care for female populations. **Methods:** The PRISMA-SCR (Preferred Reporting Items for Systematic reviews and Meta-Analyses extension for Scoping Reviews) was used as a reference checklist throughout this study. The Arksey and O’Malley methodology was used as a framework to guide this review. The framework methodology consisted of five steps: (1) Identify research questions; (2) Search for relevant studies; (3) Selection of studies relevant to the research questions; (4) Chart the data; (5) Collate, summarize, and report the results. **Results:** In the field of breast cancer risk detection and prevention, the following AI techniques and models have been applied: Machine and Deep Learning Model (ML-DL model) (*n* = 1), Academic Algorithms (*n* = 2), Breast Cancer Surveillance Consortium (BCSC), Clinical 5-Year Risk Prediction Model (*n* = 2), deep-learning computer vision AI algorithms (*n* = 2), AI-based thermal imaging solution (Thermalytix) (*n* = 1), RealRisks (*n* = 2), Breast Cancer Risk NAVIgation (*n* = 1), MammoRisk (ML-Based Tool) (*n* = 1), Various MLModels (*n* = 1), and various machine/deep learning, decision aids, and commercial algorithms (*n* = 7). In the 11 included studies, a total of 39 barriers to AI applications in breast cancer risk prediction and screening efforts were identified. The most common barriers in the application of innovative AI tools for breast cancer prediction and improved screening rates included lack of external validity and limited generalizability (*n* = 6), as AI was used in studies with either a small sample size or datasets with missing data. Many studies (*n* = 5) also encountered selection bias due to exclusion of certain populations based on characteristics such as race/ethnicity, family history, or past medical history. Several recommendations for future research should be considered. AI models need to include a broader spectrum and more complete predictive variables for risk assessment. Investigating long-term outcomes with improved follow-up periods is critical to assess the impacts of AI on clinical decisions beyond just the immediate outcomes. Utilizing AI to improve communication strategies at both a local and organizational level can assist in informed decision-making and compliance, especially in populations with limited literacy levels. **Conclusions:** The use of AI in patient education and as an adjunctive tool for providers is still early in its incorporation, and future research should explore the implementation of AI-driven resources to enhance understanding and decision-making regarding breast cancer screening, especially in vulnerable populations with limited literacy.

## 1. Background

Breast cancer incidence rates among women have slowly increased per year by 0.5% [1,2]. It is the most diagnosed cancer worldwide, surpassing even lung cancer, accounting for 31% of estimated newly diagnosed cancer cases and 15% of estimated deaths [2,3]. In 2020, breast cancer accounted for an estimated 2.3 million cases and 685,000 deaths [3]. Mainly, breast cancer has posed significant global health challenges, with notable disparities in survival rates among socioeconomically disadvantaged women [4,5]. The incidence rates vary widely among countries, with developed nations like the UK and the USA witnessing high rates due in part to an increased prevalence of risk factors and “more extensive use of mammography screening since the 1980s” [3]. Disparities in health outcomes further complicate the global burden of cancer. For instance, non-Hispanic Black women have a higher mortality rate regarding breast cancer compared to their non-Hispanic White counterparts, and this effect might be more pronounced due to specific social determinants of health such as race, socioeconomic status, and healthcare access [6,7]. The situation is exacerbated in developing countries, where globalization and economic growth are predicted to significantly increase breast cancer incidence by 2040 [8]. In India, urban areas report the highest incidence in the 40–49 age group, contrasting with rural areas where the peak is between 65 and 69 years [8].

Screening remains a pivotal strategy in early cancer detection [3]. The WHO and the American Cancer Society have set guidelines for mammography-based screenings, emphasizing their importance for women in specific age groups [8,9,10]. The US Preventive Services Task Forces (USPSTF) recommends that women between 50 and 74 years old receive a mammogram every two years, while women between 40 and 49 years old should make an individualized decision [11,12]. Although breast cancer screening aims at early detection, intrinsic limitations do exist, such as false-positive detections leading to overdiagnosis, unnecessary costs, and negative mental and health well-being [13,14]. The recent COVID-19 pandemic has further strained global cancer care and contributed to disruptions leading to potential delays in breast cancer detection, with countries such as Canada projecting significant increases in advanced-stage diagnoses and related deaths due to screening pauses [10]. As the world grapples with these challenges, the primary goal remains clear: improving cancer screening behaviors through evidence-based strategies to reduce the global cancer burden [9]. One of these strategies is the application of innovative artificial intelligence (AI) models and techniques to predict factors that contribute to informed decision-making about breast cancer screening in at-risk women [15].

AI applications within society are highly prevalent and are beginning to grow substantially within the healthcare field [15,16]. Specifically, radiology and pathology specialties are witnessing the introduction of digital workflows and AI, which offer promising prospects in the field of precision medicine. AI is a broad term that illustrates the concept of “mimicking human intelligence using computers” [17]. Computer programmers create an algorithm, and eventually, the computers can use specific data provided by programmers to make decisions [18]. AI systems and techniques have rapidly evolved over the last 20 years, transitioning from machine learning (ML) to deep learning (DL), to the inclusion of advanced pathways for imaging analysis by allowing healthcare providers to analyze spatial and contextual information from images through multiple layers and convolutional operations. When it comes to daily application of AI systems, radiologists are more effectively managing workflows and detecting suspicious lesions more accurately. Hence, certain AI systems are exceeding human capabilities in predicting long-term breast cancer risk through the development of risk scores tailored for early detection of the disease and adequate intervention.

More exposure to new information improves the ability to interpret data and make decisions [18]. Many cancer screening programs, such as breast cancer, focus on a “one size fits all” approach while prone to inter-observer variability, making patient selection and risk stratification challenging [17,18]. In addition, overdiagnosis and false positives, as previously mentioned, are concerns within the cancer screening process, which could lead to unnecessary treatment and harm to patients [19,20]. AI techniques and models can be applied in cancer prevention and management by using patient history, scans, imaging information, and analysis of specific genes for cancer classification to reduce overdiagnosis and overtreatment [21,22]. This approach can help personalize medicine and benefit all patients, providers, and the healthcare system by providing risk assessment, early cancer detection, diagnosis and classification of cancers, treatment response prediction and efficacy, and helping radiologists process a large amount of data quickly [17,18]. Additionally, there is a need to identify and understand the current circumstances of AI’s application on breast cancer screening and prevention among adults, primarily female adults, for more effective cancer care prevention and recommendations for its future use [23].

This scoping review aims to (1) compare the major outcomes from the application of the different AI models in risk score development and screening rates changes; (2) identify the barriers encountered in applying innovative AI models and techniques in promoting breast cancer screening behaviors and predicting the risk of developing breast cancer among adult females; and (3) highlight recommendations for the adoption, adaptation, and practical implementation of such tools for breast cancer risk score development and incorporation in breast cancer screening efforts. Findings from this review can inform and guide future global recommendations for AI application in breast cancer prevention and care for female populations.

## 2. Methods

The PRISMA-SCR (Preferred Reporting Items for Systematic reviews and Meta-Analyses extension for Scoping Reviews) was used as a reference checklist throughout this study [24]. The Arksey and O’Malley methodology was used as a framework to guide this review [25]. The framework methodology consisted of 5 steps: (1) Identify research questions; (2) Search for relevant studies; (3) Selection of studies relevant to the research questions; (4) Chart the data; and (5) Collate, summarize, and report the results.

### 2.1. Step 1: Identify Research Questions

The two research questions for the scoping review were: (1) What are the barriers encountered in the application of innovative AI techniques and models in promoting breast cancer screening and predicting breast cancer risk among adult females worldwide? And (2) what are global future recommendations for AI application in breast cancer prediction and early detection for female populations?

### 2.2. Step 2: Search for Relevant Studies

Keywords and mesh terms were developed by a research librarian (MK) experienced with scoping review protocols to allow for the replication of the methodology used for future reviews and other studies relevant to the topic at hand (Supplementary File S1). Search terms included AI, ML, clinical decision aid, computational intelligence, machine computer reasoning, breast cancer, breast neoplasm, malignant tumor of the breast, screening, pre-screening, early detection, model prediction, breast cancer risk, and risk score. The Rayyan platform was used to condense all studies generated from searching four electronic databases (PubMed, Embase, Web of Science, and Cochrane Library) [26]. The review of the literature was conducted over a two-month period from September 2023 to November of 2023. Screening of the articles for inclusion was carried out by primary author (LS) and co-authors (DL, SB, KL, EM, NG, JX, RM, GS). 

#### 2.2.1. Inclusion Criteria

Included articles were peer-reviewed studies that were published in English between 2013 and 2023 that (1) examined machine learning and artificial intelligence software and models designed to predict breast cancer risk and/or promote breast cancer screening measures in adult women globally, and (2) explored the role of artificial intelligence and/or machine learning in improving breast cancer screening rates and early detection measures in adult women. AI software and models encompassed all AI techniques such as machine learning, deep learning, robotics, data mining, and reasoning that were specifically designed to predict breast cancer risk in adult women based on social determinants of health, genetic and environmental factors, and other components rendering these women at-risk of developing the disease at one point in their life. Studies were also included if these AI models and techniques were used to influence screening behavior to improve breast cancer screening rates and impact of early detection and prevention efforts in the at-risk female population at a global level.

#### 2.2.2. Exclusion Criteria 

Studies were excluded if they (1) addressed cancers other than breast cancer, (2) were not focused on AI, the application of an innovative AI model, technique, or methodology, (3) targeted both male and female patients, (4) included female patients under the age of 18, and (5) were not written in English. Finally, studies that were published as abstracts or used a systematic, scoping, or narrative review methodology were excluded.

### 2.3. Step 3: Selection of Studies Relevant to the Research Questions

Initial article screening, extraction from the relevant databases, and Rayyan page construction were performed by the lead author (LS). Co-authors (DL, SB, KL, EM, NG, JX, RM, GS) conducted a secondary screening of titles and abstracts in pairs (KL and GS; RM and JX; DL and NG; SB and EM). Consensus on disagreements was reached via discussion involving the initial reviewer (LS).

Co-authors (DL, SB, KL, EM, NG, JX, RM, GS) extracted, summarized, and tabulated the data from all relevant studies. Senior author (LS) reviewed all tabulated data to resolve any discrepancies. Summary tables included one evidence table describing study characteristics (Table 1). Table 2 summarized the barriers encountered in the application of innovative AI techniques and models in promoting breast cancer screening and/or predicting breast cancer risk among adult females globally. Table 3 provides future directions and recommendations in building more effective models for increased accuracy in breast cancer risk prediction and early detection of the disease through the promotion of screening behaviors in adult women. Basic qualitative content analysis was carried out to identify similar themes in recommendations for the advancement of AI models and techniques for breast cancer risk prediction and increased effective screening measures across studies.

### 2.4. Steps 4 and 5: Data Charting, Collation, Summarization, and Reporting of Results

Study characteristics were tabulated for primary author, year of publication, study design, country, sample size, study population, study purpose, type of AI model or technique applied to breast cancer screening/risk prediction, and major outcomes (Table 1). Common limitations and challenges in the application of AI techniques and models were highlighted across the included studies (Table 2). For Table 3, the three phases of qualitative content analysis for the results of primary qualitative research described by Elo and Kyngas (2008) were applied: (i) preparation, (ii) organizing, and (iii) reporting [38]. In the preparation phase, the unit of analysis is selected, which in our case was relevant lessons learned from each of the included studies in the application of AI techniques and models. This is followed by the organizing phase which encompasses data coding, grouping, categorization, and abstraction of lessons learned across studies for theme identification. The final phase, reporting, consists of sharing the results from the analysis process through tabulated categories. 

Content analysis allows the description of the phenomenon in a conceptual form. For the purpose of our paper, deductive analysis was carried out since the resulting structure of the qualitative analysis was operationalized based on previous knowledge in the included studies. Additionally, a deductive approach allowed us to compare theme categories at different time periods of the published studies [38]. This methodology has been widely used in the initial assessment of innovative approaches in healthcare studies [38] and aided in the identification of recurrent themes in recommendations for future advancements in the application of AI to prevent and screen for breast cancer.

## 3. Results

The initial study extraction yielded 5814 results from PubMed (*n* = 3054), EMBASE (*n* = 1455), Web of Science (*n* = 1245), and Cochrane (*n* = 60). A total of 2730 duplicate studies were excluded (*n* = 1226 from PubMed, *n* = 1344 from Embase, *n* = 103 from Web of Science, and *n*= 57 from Cochrane). A total of 3084 studies were screened for eligibility by review of their abstracts. A total of 3070 articles were excluded due to focus on breast cancer diagnosis, treatment, malignancy detection, tumors, or breast density rather than on breast cancer risk detection or screening initiation (*n* = 1954), lack of artificial intelligence application (*n* = 592), wrong population (*n* = 373), wrong study design (*n* = 144), and publication in a language other than English (*n* = 7). Fourteen studies were initially selected for full text review and were sourced from PubMed (*n* = 7), EMBASE (*n* = 3), Web of Science (*n* = 1), and Cochrane (*n* = 3). Upon full article review, three studies were excluded due to being published as abstracts without full texts. 

**Figure 1:**
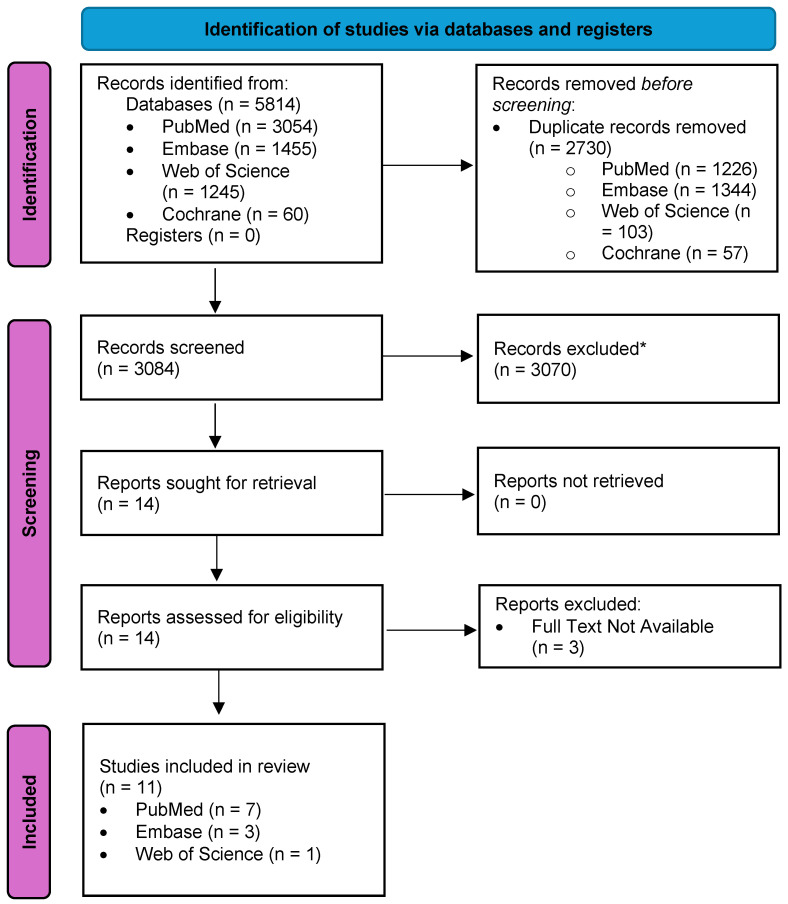
PRISMA Flow Diagram of the Study Selection Process. Reasons for record exclusion (*) were as follows: Wrong Outcome including focusing on breast cancer diagnosis, breast cancer treatment, detection of malignancy, tumors, or breast density, not focusing on breast cancer risk detection, screening initiation, and mammography (*n* = 1954); No application of artificial intelligence tools (*n* = 592); Wrong Population (*n* = 373); Wrong Study Designs including systematic review, scoping review, narrative review, and meta-analysis (*n* = 144); Published in a Language Other Than English (*n* = 7).

A total of eleven studies were retained for full analysis [27,28,29,30,31,32,33,34,35,36,37], including three retrospective case-cohort studies (*n* = 3), three retrospective cohort studies (*n* = 3), two mixed-methods studies (*n* = 2), and one randomized controlled trial (*n* = 1), pilot usability study (*n* = 1), and feasibility study (*n* = 1). All 11 retained studies were published between 2015 and 2023 and had sample sizes ranging from *n* = 6 to *n* = 64,739. The study selection and review process are detailed in Figure 1.

In the field of breast cancer risk detection and prevention, the following AI machine learning tools have been applied: Machine and Deep Learning Model (ML-DL model) (*n* = 1), Academic Algorithms (e.g., Mirai Algorithm, Globally Aware Multiple Instance Classifier Algorithm (*n* = 2), Breast Cancer Surveillance Consortium (BCSC), Clinical 5-Year Risk Prediction Model (*n* = 2), deep-learning computer vision AI algorithms (e.g., Mirai, MammoScreen, ProFound AI, Mia, GMIC) (*n* = 2), AI-based thermal imaging solution (Thermalytix) (*n* = 1), RealRisks (*n* = 2), Breast Cancer Risk NAVIgation (*n* = 1), MammoRisk (ML-Based Tool) (*n* = 1), Various MLModels (*n* = 1), and various machine/deep learning, decision aids, and commercial algorithms (e.g., Logistic Regression, Naive Bayes, Decision Tree, Support Vector Machine, Linear Discriminant Analysis, Neural Networks) (*n* = 7).

### 3.1. Major Outcomes

The ML-DL model used by Akselrod-Ballin et al. predicted breast malignancy, identified false negative findings from previous mammograms, and outperformed existing clinically based risk models. When combined with clinical risk models, these ML-DL models improved predictive performance. Meanwhile, a variety of academic and commercial algorithms demonstrated significantly higher sensitivity in predicting breast cancer risk compared to traditional radiology assessments and higher discrimination than the BCSC model (*n* = 2). Additionally, the combined use of AI algorithms and the BCSC clinical risk model marked a significant increase in predictive accuracy (*n* = 2). This combination also reduced over-screening and under-screening of patients. They also showed improved performance when trained for shorter periods (*n* = 2). Furthermore, an ML model using clinical data showed improved model performance over time and helped identify novel clinically relevant patterns. The AI-based thermal imaging solution, Thermalytix, was effective in breast cancer screening. Decision aids such as RealRisks improved accuracy in breast cancer risk perception. MammoRisk, an ML-based tool, influenced changes in risk scores and screening decisions based on polygenic risk scores (PRS). The application of various ML models, including logistic regression and neural networks, provided diverse insights contributing to breast cancer risk detection and prevention. In summary, these AI techniques and models have significantly contributed to enhancing the accuracy of breast cancer risk detection and mammography screening, demonstrating the potential for improved early detection and patient-specific screening strategies (Table 1). 

### 3.2. Common Barriers in AI Applications for Breast Cancer Prediction and Prevention

In the 11 included studies, a total of 39 barriers to AI applications in breast cancer prediction and prevention were identified (Table 2). The most common barriers in the application of innovative AI techniques and models to promote breast cancer screening behavior and improve breast cancer risk detection included lack of external validity and limited generalizability (*n* = 6), as AI was used in studies with either a small sample size or datasets with missing data. Many studies (*n* = 5) also encountered selection bias due to the exclusion of certain populations based on characteristics such as race/ethnicity, family history, or past medical history. In regard to challenges encountered by healthcare providers in the application of such tools and models, financial limitations (*n* = 2) and negative attitudes of female patients toward screening services (*n* = 2) additionally impacted the role of AI in breast cancer screening. The breast cancer risk models commonly demonstrated high levels of uncertainty (*n* = 3) and could not estimate when women should begin screening (*n* = 1). They also faced limitations with technical aspects, such as graphic demand (challenges in producing high-quality quantitative image feature analysis-based prediction models) (*n* = 1), long-term outcome prediction (*n* = 1), precise localization (*n* = 1), and differentiation between calcification and mass (*n* = 1). After receiving the results, patients had only a limited understanding of them, in part due to their complexity (*n* = 1). Finally, screening risk with underestimation could impact patient future decision making, and decreasing follow up (*n* = 1) (Table 2).

### 3.3. Lessons Learned and Future Directions

There are many considerations and future directions for the application of AI techniques and models for breast cancer screening (Table 3). First, AI models urgently need to include a broader spectrum and more complete predictive variables for risk assessment. Hence, there is a need to invest in generating diverse datasets to enhance the practicality and validity of AI models for breast cancer screening. Second, investigating long-term outcomes with improved follow-up periods is critical in assessing the impacts of AI on clinical decisions beyond just the immediate outcomes. Third, to enhance external validity, there are avenues for improvement, such as addressing issues with incomplete variable datasets and small sample sizes that could impact the accuracy of findings, along with including diverse population groups to avoid selection bias and ensure the generalizability of AI models. Fourth, personalized risk assessments and screenings are essential for cancer prevention strategies and can be improved by expanding datasets to include a broader spectrum of predictive variables for risk assessment. Fifth, to increase general applicability, the models should be validated with other populations to address potential biases related to race/ethnicity, family history, or past medical history that might arise in dataset selection. Finally, utilizing AI to improve communication strategies at both a local and organizational level can assist in informed decision-making and compliance, especially in populations with limited literacy levels. A concise set of variables such as benefits, harms, and statistics need to be present and clear to provide the opportunity for informed decision-making on breast cancer screening and improve patient-provider communication on the role of AI models in breast cancer risk prediction [27,28,29,30,31,32,33,34,35,36,37].

## 4. Discussion

The purpose of this scoping review was to identify the obstacles encountered when applying current innovative AI techniques and models to foster breast cancer screening behaviors and improve breast cancer risk prediction among adult females. As AI techniques and models continuously evolve in nature and scope, they are more equipped to predict breast cancer risk in women accurately [39,40,41]. Romanov et al.’s model received an AUC of 0.747 when predicting cancer-free mammograms from women who went on to develop breast cancer with high predictive power, while the Mirai model maintained its accuracy across seven different populations across five countries [39,40]. By incorporating diverse demographics into AI algorithms, AI offers the opportunity to individualize care and reduce healthcare disparities, such as racial and socioeconomic bias, and allow healthcare to become more equitable [40,42]. Its application in early breast cancer risk detection has shown advantages towards breast cancer screening adherence by encouraging short-term and long-term actions among women [37]. For instance, women who receive high-risk estimates for breast cancer could potentially be motivated to seek a physician early to begin screening and take preventative actions, like hormone therapy replacement or chemoprevention, before breast cancer arises [37]. Some AI models have been designed to also identify those women at high risk for poor psychological resilience after breast cancer diagnosis, to provide early resources to women most in need to improve mental health and quality of life in the future [43]. In addition, AI can serve as a feasible and affordable option, especially in promoting healthcare access in underserved and resource-poor populations [31,37]. At the organizational level, implementing AI models can reduce the burden on the healthcare system, as demonstrated by Ng et al.’s study, which noted a 45% workload reduction while still enhancing breast cancer detection [44]. Overall, the incorporation of AI alongside physicians has been shown to significantly reduce diagnostic time and enhance diagnostic accuracy, ultimately providing efficiency within the workplace [45,46,47].

It is crucial to address the major barriers that limit the worldwide implementation of AI models for improving breast cancer screening rates and early detection through risk scores. One significant barrier identified from this review is the limited generalizability of AI models due to small sample sizes or incomplete variable datasets. These limitations are frequently reported and stem from the challenges associated with gathering large, diverse, and comprehensive datasets that accurately reflect the broader population [48]. The issue underscores the need for additional funding to support the collection of data that accurately represents diverse populations globally, ensuring inclusivity in risk assessment models and their application across larger and more diverse sample sizes [49,50]. Moreover, integrating social determinants of health (SDOH) into developed risk scores is imperative to ensure these tools are more inclusive and accurately represent the populations at risk [51]. SDOH encompasses a range of factors, such as socioeconomic status, education, neighborhood and physical environment, employment, and social support networks, as well as access to healthcare [52]. By incorporating these factors into AI-driven risk assessments, models can provide a more nuanced and comprehensive evaluation of an individual’s risk for developing breast cancer [53]. This approach not only enhances the precision of risk scores but also addresses the disparities in healthcare access and outcomes among different demographic groups, particularly underserved and minority populations, by taking into consideration the underlying factors contributing to increased breast cancer risk in such racial and ethnic groups [51,52,53]. In addition to challenges related to data diversity, model generalizability, and the integration of comprehensive risk factors, this review found that financial limitations and technical challenges hinder the potential of AI and ML tools to revolutionize breast cancer screening and early detection. Overcoming these obstacles requires concerted efforts to secure additional funding for staff training on effective application of AI models, foster collaborative research initiatives, and develop methodologies for integrating SDOH into AI models, thereby ensuring that these innovative tools can benefit a wider range of populations globally and contribute to the reduction of breast cancer morbidity and mortality [51,52,53].

It has been established throughout this review that the incorporation of AI into breast cancer screening is a promising tool for early detection and improved outcomes, but it also highlights the need for a multi-level approach when discussing ML to enhance general applicability and validity across numerous sociodemographic groups. Some literature has shown AI-based models to be accurate among diverse datasets; however, there remains a need for training these models on more robust, diverse datasets [39]. The existing literature trains these models on large datasets, with hundreds to thousands of patients, yet these often come from a single study site and/or community [40,41]. Research around AI and breast cancer screening needs to invest in generating more diverse datasets to elevate these proof-of-concept models by improving their practicality and reducing data bias. With this said, generating a diverse dataset can be challenging. Shams et al. investigated diversity and inclusion within AI research and found that studies point out an under-representation of minority groups in sampling during model training/testing, that there is less attention on equity and justice in AI design and development in general, and there is a general difficulty in measuring diversity within an algorithm [54]. An obvious solution to overcome this is to share data across institutions, but this becomes highly implausible due to patient privacy policies. To circumvent this, researchers could share their models while data remains local to the study site, the concept of federated learning, to further develop their algorithms [55]. Until minority groups are considered in the design, development, and implementation of AI systems, these groups are potentially not receiving any benefit from such technologies [54,55].

Moreover, underserved minority groups often face barriers to informed decision-making due to limited healthcare resources and lower health literacy rates [56,57]. Consequently, lower health literacy rates are associated with numerous poor health and behavioral outcomes [58]. Notably, individuals with inadequate self-reported health literacy have been found less likely to be adherent to mammography guidelines and have been associated with increased cancer fatalism [59,60]. This highlights a clear deficit in how health-related information is presented or communicated [61]. AI can play a pivotal role in developing an improved decision-making aid on breast cancer screening in underserved communities. The release of more widely available AI platforms, such as OpenAI’s ChatGPT and Google’s Bard, has increased public consciousness about AI, with one study finding 80% of Americans willing to use AI-power tools in their health management, underscoring its potential uses in public health across a broad community [62]. Some of these conversational chatbots have already been investigated in cancer screening, prevention, and management with some success [63,64]. Beyond chatbots, AI can be applied in redesigning existing patient education materials to different reading levels for patients [65]. The use of AI in patient education and as an adjunctive tool for providers is still early in its incorporation, and future research should explore the implementation of AI-driven resources to enhance understanding and decision-making regarding breast cancer screening, especially in vulnerable populations with limited literacy [61,62,63,64,65].

If these concerns are not addressed within the domain of breast cancer screening development, late-stage diagnosis continues to be a considerable burden for patients and their providers. To add to this, breast cancer screening has declined due to the COVID-19 pandemic in the US [23]. Women from racial/ethnic minorities (i.e., American Indian/Alaskan Native, Asian/Pacific Islander, Hispanic) and rural areas particularly are seeing great declines in screening test rates [23]. This recently observed trend may consequently result in delayed identification and late-stage disease diagnosis. AI can potentially step in by improving patient education and risk assessment/stratification, as well as contribute to a more automated, cost-effective approach that would hopefully enhance existing and develop new screening and diagnostic approaches as it relates to breast cancer, benefiting both healthcare providers and at-risk women [19,20,21,22,23].

### Limitations

Findings from this review should be interpreted in the context of study limitations. Although a comprehensive search across four databases was carried out for article selection that are relevant to our inclusion criteria, this review did not include tracing of reference lists, manual searches of journals, or grey literature. Additionally, this review only focused on breast cancer screening and risk prediction measures, and excluded breast cancer diagnostic measures. Broader reviews are recommended to account for other sources of literature and extend to diagnostic and management measures rather than focusing solely on preventive measures. Second, artificial intelligence in healthcare is a rapidly evolving field, so it is possible that some studies were not included due to the unintentional omission of search terms. Collaboration with a research librarian for a thorough development of mesh terms to include technical keywords relevant to machine learning and artificial intelligence has likely mitigated this concern. Third, since this is a scoping review, a formal assessment of the quality of the included studies was beyond the scope of this paper. Future systematic reviews should apply a validated checklist from the AI field to adequately assess the application and limitations of the diverse AI tools in predicting breast cancer and promoting screening behavior. 

## 5. Conclusions

This scoping review describes efforts to apply innovative AI techniques and models to improve breast cancer screening rates and enhance the accuracy of breast cancer risk prediction scores. Results may contribute to a broader understanding of the limitations of these tools in breast cancer screening and breast cancer prevention measures, particularly in developing countries with limited affordability and quality of such innovative resources. This study can inform future AI healthcare specialists on more effective ways to improve the global reach and sustainability of these tools in underserved female communities who are at-risk of developing breast cancer.

## Figures and Tables

**Table 1:** Study Characteristics.

Article #	Primary Author/Year	Study Design	Country	Sample Size	Study Population	Study Purpose	Type of AI Model/Technique Applied to Breast Cancer Screening and/or Risk Prediction	Major Outcomes
1	Akselrod-Ballin et al., 2019 [27]	Retrospective Cohort Study	Israel	*n* = 13,234	Women who underwent at least one mammogram between 2013 and 2017 in one of the five Assuta Medical Centers imaging facilities, and who had health records for at least 1 year before undergoing mammography in Maccabi Health Services	To evaluate the accuracy and efficiency of a combined machine and deep learning approach for early breast cancer detection applied to a link dataset of digital mammography images and detailed electronic health records	- Machine and deep learning model (ML-DL model)—prediction model	- Predicted breast malignancy detected within 12 months from the index examination - Algorithm identified false negative findings missed by radiologist - Compared with existing clinically based risk models, prediction with clinical data alone outperformed the Gail - The ML-DL models that combined information from both images and clinical data performed better than images or clinical data alone
2	Arasu et al., 2022 [28]	Retrospective Case-Cohort Study	USA	*n* = 13,881	Women who had a bilateral screening mammogram in 2016 at Kaiser Permanente Northern California, without evidence of cancer on final imaging assessment either at the time of screening or after diagnostic work up of positive screening findings	To examine the ability of 5 artificial intelligence (AI)-based computer vision algorithms, most trained to detect visible breast cancer on mammograms, to predict future risk relative to the Breast Cancer Surveillance Consortium clinical risk prediction model (BCSC v2)	- Academic algorithms (Mirai algorithm, Globally Aware Multiple Instance Classifier algorithm) Commercial algorithms (anonymous) - Breast Cancer Surveillance Consortium (BCSC) clinical 5-year risk prediction model	- All AI algorithms had significantly higher discrimination than the BCSC clinical risk model for predicting 5-year risk - AI algorithms trained for short time horizons can predict risk of cancer up to 5 years when no cancer is clinically detected on mammography - Combined AI and clinical risk model marginally improved performance compared with any AI model alone - The combined model also decreased overall differences in discrimination between AI algorithms - Continued strong predictive performance up to 5 years suggests AI is no longer identifying missed cancers, but features of true underlying risk
3	Arasu et al., 2023 [29]	Retrospective Case-Cohort Study	USA	*n* = 13,628	Women who had a bilateral screening mammographic examination in 2016 at Kaiser Permanente NorthernCalifornia that was negative at final imaging assessment, and were followed until 2021	To compare selected existing mammography artificial intelligence (AI) algorithms and the Breast Cancer Surveillance Consortium (BCSC) risk model for prediction of 5-year risk	- Five deep-learning computer vision AI algorithms: Mirai, MammoScreen, ProFound AI, Mia, GMIC - Breast Cancer Surveillance Consortium (BCSC) clinical risk model	- AI algorithms performed better than the BCSC risk model for predicting breast cancer risk at 0 to 5 years - AI algorithms trained for short time horizons can predict future risk of cancer up to 5 years when no cancer is clinically detected at mammography - Combined AI and BCSC models further improved prediction
4	Chorev et al., 2023 [30]	Retrospective Cohort Study	Israel	*n* = 13786 (Israel); *n* = 1695 (US)	Israeli and American women who underwent screening mammography	To assess the utility of a personalized breast cancer (BC) risk model using comprehensive health records	- Machine learning model using clinical data	- Improved performance of the model over previous BC risk models - Identification of novel clinically relevant risk factors
5	Davalagi et al., 2022 [31]	Mixed-Methods Study	India	*n* = 768	Women in the reproductive age group from urban slums of central Karnataka, India	To assess the acceptance and explore challenges for an AI-based screening solution for breast health among the urban slum population	- AI-based thermal imaging solution (Thermalytix)	- The AI-based screening was found to be a feasible solution for breast health in low-income, low health access areas like urban slums
6	Hersch et al., 2015 [32]	Randomized Controlled Trial	Australia	*n* = 879	Women aged 48–50 years from New South Wales, Australia	To investigate the impact of including information about breast cancer over detection in a decision aid on informed choice in breast screening	- Decision aid including information on breast cancer over detection	- Women who received the decision aid with over detection information were more likely to make an informed choice about breast screening
7	Kukafka et al., 2015 [33]	Mixed-Methods Study	USA	*n* = 34	Multi-ethnic women from Upper Manhattan, predominantly Hispanic, with a high proportion of low numeracy	To evaluate a decision aid, RealRisks, in improving breast cancer risk perception and decision-making in low-numerate women	- Web-based decision aid, RealRisks	- Significant improvement in accuracy of perceived breast cancer risk after using RealRisks
8	McGuinness et al., 2022 [34]	Pilot Usability Study	USA	*n* = 6	EHR data of 6 patient advocates	To evaluate whether the Fast Healthcare Interoperability Resources (FHIR) standard could support automated breast cancer risk calculations in RealRisks and Breast cancer risk NAVIgation (BNAV) as well as presentation of relevant patient medical history to patients and providers to facilitate shared decision-making	- RealRisks and Breast cancer risk NAVIgation	- Certain categories of patient data—particularly gynecologic history, family history of cancer, and history of genetic counseling and testing—were documented less frequently than other data types or not at all—highlights the limitations of using EHR data alone in risk calculation - EHR data from Fast Healthcare Interoperability Resources (FHIR) could be incorporated into automated breast cancer risk calculation in clinical decision support tools
9	Portnoi et al., 2019 [35]	Retrospective Cohort Study	USA	*n* = 1656	High-risk women who were screened for breast cancer due to risk factors: genetic mutation, chest radiation, family history of breast cancer, or personal history of breast cancer	To develop a deep learning-based model to analyze breast MR and predict 5-year breast cancer risk	- Deep Learning	- When compared to the Tyrer-Cuzick Model, the deep learning model had a statistically significant greater AUC value. The researchers also developed a risk factor regression model based on traditional risk factors, which did not have a significantly different AUC
10	Saghatchian et al., 2022 [36]	Feasibility Study	France	*n* = 196	Women aged 40 or older, primarily of Caucasian origin, undergoing breast cancer risk assessment	To assess the feasibility of personalized screening and prevention recommendations in the general population through breast cancer risk assessment at a dedicated risk clinic	- MammoRisk, a machine learning-based tool	- PRS changed the risk score and screening recommendations in 40% of women; 28% shifted from intermediate to moderate or high risk
11	Stark et al., 2019 [37]	Retrospective Cohort Study	USA	*n* = 64,739	Study population was derived from the PLCO (Prostate, Lung, Colorectal, and Ovarian) cancer screening trial data, focusing on women who self-identified as White, Black, or Hispanic	To predict breast cancer risk using personal health data and machine learning models	- The study applied several machine-learning models, including logistic regression, naive Bayes, decision tree, support vector machine, linear discriminant analysis, and neural networks	- Various results of this study contribute to understanding the feasibility and effectiveness of using machine learning models for breast cancer risk prediction and how they compare with traditional models like BCRAT - The developed model could be used as non-invasive and cost-effective risk stratification tools to increase early breast cancer detection and prevention, motivating both immediate actions like screening and long-term preventative measures such as hormone replacement therapy and chemoprevention

**Table 2:** Common Barriers in AI application for Breast Cancer Prediction and Prevention.

Article #	Primary Author/Year	Barriers	Common Barriers in AI Application for Breast Cancer Screening and Risk Prediction
1	Akselrod-Ballin et al., 2019 [27]	Selection bias: Exclusion of women with a personal history of breast cancer lead to general cohort having lower number of relatives in breast cancer family history of women with biopsy positive for cancer Used images from one mammography vendor. Due to the process by which data was transfer, women were excluded on the basis of a single nonmalignant DM examination without follow up while those with benign findings were included Screening and diagnostic studies was not well defined Model does not yet offer a localization of the finding, only global probability Do not have data to differentiate between findings like calcification or mass	Lack of external validity and limited generalizability due to incomplete variable datasets and small sample size Selection bias by excluding certain populations (i.e., race/ethnicity, family history, past medical history) Financial barriers with screening services Screening risk might impact future decision making, leading to reduced services and follow up Uncertainty of breast cancer risk models (overestimation or underestimation) Not well defined when women should begin screening Negative attitudes toward breast cancer screening and health systems Risk models may lack technical considerations (i.e., long-term outcomes predictions, graphic demand, precise localization, or differentiation between calcification and mass) Limited understanding of breast health or complexity of results by general population Potentially limited resources within facilities
2	Arasu et al., 2022 [28]	Retrospective ascertainment of BCSC clinical risk model inputs for family history and prior breast biopsies could have led to underestimation of BCSC performance Most algorithms have not been trained to predict long term outcomes	
3	Arasu et al., 2023 [29]	Mirai was originally calibrated for both diagnostic and future risk, leading to overestimated cancer risk AI risk models are limited to women who have undergone mammography, so cannot inform when a women should start screening. AI risk models also have potential costs and other technical and workflow considerations for implementation. Unable to evaluate all existing mammography AI algorithm Unable to assess the extent to which family history was missing	
4	Chorev et al., 2023 [30]	Variability in clinical data available in different facilities Need for models to be adaptable to different racial/ethnic groups Incomplete data collection and variety in mammography workstations in the American dataset Generalization issue of AI models across different populations	
5	Davalagi et al., 2022 [31]	Poor breast health awareness among women Reluctance to follow-up for further evaluation in asymptomatic women Non-availability of ultrasound-guided Fine Needle Aspiration Cytology (FNAC) at the screening center Cost associated with travel to tertiary care center Lack of volunteers for house-to-house visits for screening Fear of results prevented women from accessing Thermalytix services Loss of daily wage	
6	Hersch et al., 2015 [32]	Complexity of conveying information about over detection and its impact on decision-making Difficulty in changing pre-existing positive attitudes towards breast screening Challenge in ensuring comprehension of statistical information by the general population	
7	Kukafka et al., 2015 [33]	Uncertainty about breast cancer risk and risk models Distrust towards the healthcare system Perception of risk assessment as a proxy for rationing access to care Financial barriers to genetic testing	
8	McGuiness et al., 2022 [34]	Small sample size of only six patients, likely introduced bias into the type of data variables that would be found (or missing) in the downloaded files, and therefore might limit the generalizability of findings	
9	Portnoi et al., 2019 [35]	Due to graphical demand by the model, 3D volume was compressed to 2D, which decreased performance	
10	Saghatchian et al., 2022 [36]	Single-center study with a small sample size Higher proportion of participants with risk factors compared to general population PRS only validated in women of Caucasian origin, The findings may not be generalizable to the broader population	
11	Stark et al., 2019 [37]	Limited predictor variables due to reliance on available data in the PLCO dataset Lack of external validation to demonstrate the generalizability of the models Potential bias due to excluding certain demographic groups based on the data available in the PLCO dataset	

**Table 3:** Lessons Learned and Future Recommendation in AI application for breast cancer screening and risk prediction efforts.

Article #	Primary Author/Year	Recommendations	Recurrent Themes for Future Directions in AI Application for Breast Cancer Screening and Risk Prediction
1	Akselrod-Ballin et al., 2019 [27]	By offering a careful cohort selection, we can avoid/adjust for biases By offering clinical centered features, physicians can transcend correlation-based predictions into causal networks of clinical factors leading to a diagnosis Potential to personalized screening methods by training machine learning algorithms on available clinical data To correct for selection bias, limit the cohort to women undergoing their first mammographic examination Results must be validated across different vendors, facilities, and populations Potentially using it as a second reader where double reading may not be available Incorporating US imaging or additional clinical data such as genetic information to improve accuracy and expert time	Need to recalibrate AI models to include more comprehensive predictive variables to access risk assessment (i.e., imaging, self-reported data, genetics) Need to look at long-term outcomes with longer follow-ups Need to increase external validity and decrease hidden biases by increasing sample size, diversifying population (i.e., race/ethnicities), and including external datasets and population-based studies Need for personalized risk assessment/screening Validating models with other populations Communication strategies, at local and organizational level, to help in informed decision-making and compliance Considering literacy level of population and proving clear, concise information (i.e., benefits, harms, statistics) to help women in breast screening decisions
2	Arasu et al., 2022 [28]	Larger gains in improvement may be derived by combining clinical risk and mammography AI with single nucleotide polymorphism polygenic risk scores Mirai model may need to be recalibrated due to overestimated cancer risk Before AI is applied, it should be evaluated in specific populations that are likely to experience health disparities if there are hidden biases in the algorithm Impact on clinical decisions requiring longer-term data, such as chemoprevention or hereditary genetic screening, requires further study in cohorts with longer follow up	
3	Arasu et al., 2023 [29]	AI models trained to predict specific thresholds can be recalibrated to support these decisions Before AI is applied, it should be evaluated in the local patient populations for validity and potential hidden biases or disparities Most of the algorithms evaluated have not yet been trained to predict longer-term outcomes, suggesting a rich opportunity for further improvement. Evaluating a larger sample of AI algorithms The impact of AI models on clinical decisions requiring risk prediction beyond 5 years requires further study in cohorts with longer follow-up	
4	Chorev et al., 2023 [30]	Consideration of different scaling for lab tests for different races/ethnicities Inclusion of more comprehensive data sources such as genetic information for improved results Need for further research in the generalization of AI models from one population to another Exploration of the interchangeability and compensation between different clinical factors in model development	
5	Davalagi et al., 2022 [31]	Engagement of local community and active involvement of health system for the sustainability of novel strategies Counseling services before and after the screening test to improve compliance Need for comprehensive approaches like AI-based imaging and increased stakeholder participation for effective breast health care in resource-constrained areas Need for an extended study of women from different strata and among PCPs to understand their perspective	
6	Hersch et al., 2015 [32]	Need for decision aids to include comprehensive information about both benefits and harms of breast screening Importance of providing clear and understandable statistical information to aid informed decision-making Further research to explore the impact of detailed information on women’s choices about breast screening	
7	Kukafka et al., 2015 [33]	Addressing the need for improved communication strategies for risk information Ensuring credible and trustworthy sources for risk information Addressing financial barriers to access genetic testing and other preventive measures Designing interventions that consider the numeracy and literacy levels of the target population	
8	McGuiness et al., 2022 [34]	A potential solution for automated risk calculation in RealRisks and BNAV decision support tools is to incorporate both self-reported data and data automatically populated using the FHIR interface	
9	Portnoi et al., 2019 [35]	Image-only deep learning models can be more accurate than traditional risk factor models, especially when risk factors are not available in the patient history, thus warranting further research and external validation	
10	Saghatchian et al., 2022 [36]	Personalized risk assessment meets a real need for individuals aware of their breast cancer risk Population-level studies are needed to assess the clinical utility of individual risk assessment Risk assessment tools need to be further investigated for performance	
11	Stark et al., 2019 [37]	Include a more diverse demographic in the study to improve the generalizability of the findings Utilize external datasets for validation of the machine learning models Explore the inclusion of more comprehensive predictor variables that might influence breast cancer risk	

## Supplementary Materials

The following supporting information can be downloaded at: https://www.mdpi.com/article/10.3390/jcm13092525/s1.
