# Supplementary material for: Promoting Artificial Intelligence for Global Breast Cancer Risk Prediction and Screening in Adult Women: A Scoping Review
Source: J Clin Med. 2024 Apr 25;13(9):2525. doi: 10.3390/jcm13092525 (PMC11084581; doi:10.3390/jcm13092525)
Supplement: Supplementary file 1 [file jcm-13-02525-s001.zip › jcm-2951626-supplementary.pdf]

Florida Atlantic University
Schmidt College of Medicine
Systematic & Scoping Review Service

Level of Service:

Collaboration: The FAU Medical Library Faculty Member will partner with you as a co-author on your review. As your co-author, the librarian will develop validated, reproducible searches for the relevant library databases in your discipline and will search the gray (non-peer-reviewed) literature for completeness. The librarian will provide you with the appropriate reference files from those searches to upload to your preferred review tool along with a draft of the search method and strategy for the methods section of the paper. The librarian can also assist you with the creation of a PRISMA flow diagram.

Librarian Collaborator or Consultant:

Michelle Keba Knecht, MSIS, MSL, AHIP - created search strategy
Tiffany Follin, MLIS - peer reviewed search strategy

Project Contact:

Dr. Lea Sacca

Type of Review:

Scoping Review

Topic/Research Question:

Title: Promoting Artificial Intelligence and Machine Learning Tools for Cancer Screening in Women
Keywords: health providers, physicians, primary care, preventive care, artificial intelligence, breast cancer, screening, mammogram, mammography, machine learning, females, women, United States
Research Questions

1. What are the barriers encountered in the application of innovative AI, machine learning, and technological tools in promoting cancer screening among adult females?
2. What is the role of patient-provider communication and trust in ensuring the success of AI application and integration in primary care and prevention efforts?
3. What are global future recommendations for AI application in cancer prevention and care for female populations?

FAU SCHMIDT COLLEGE OF MEDICINE
Florida Atlantic University

Inclusion and Exclusion Criteria:

Inclusion Criteria:
- 2013-2023
- Address cancers affecting adult women globally
- Explore the role of artificial intelligence and/or machine learning in improving cancer screening rates in adult women

Exclusion Criteria:
- Not focused on cancers affecting women
- Not focused on artificial intelligence, application of innovative technological tools, or machine learning methodology
- Targeting both males and females

Benchmark Articles:

Instructions: Input the PMIDs of the benchmark articles into the Yale MeSH Analyzer https://mesh.med.yale.edu/ to identify MeSH terms for the search term chart below. After you have created the search, test to make sure the search is pulling each of the benchmark articles by combining the search strategy AND the PMID.

https://www.sciencedirect.com/science/article/pii/S0738399113001663
https://link.springer.com/article/10.1186/s12911-022-02058-3
https://pubs.rsna.org/doi/full/10.1148/radiol.2018181371

- 23664232
- 36476612
- 30457482

FAU SCHMIDT COLLEGE OF MEDICINE
Florida Atlantic University

Search Term Chart:

Keywords	MeSH Terms (PubMed/MEDLINE)	MeSH entry terms (PubMed), Emtree Synonyms (Embase), and other synonyms	Emtree Terms (Embase)
Artificial intelligence	Decision support systems, clinical<br>Artificial intelligence<br>Image Processing, Computer-Assisted	Artificial intelligence<br>Clinical decision aid*<br>Decision aid*<br>Machine learning<br>Learning, machine<br>Intelligence, Artificial<br>Computational Intelligence<br>Intelligence, Computational<br>Machine Intelligence<br>Intelligence, Machine<br>Computer Reasoning<br>Reasoning, Computer<br>Computer Vision System*<br>System, Computer Vision<br>Systems, Computer Vision<br>Vision System, Computer<br>Vision Systems, Computer<br>Clinical Decision Support*<br>Decision Supports, Clinical<br>Support, Clinical Decision<br>Supports, Clinical Decision<br>Decision Support, Clinical	Artificial intelligence<br>clinical decision support system<br>Image processing
Breast cancer	Breast Neoplasms	Breast Neoplasm<br>Neoplasm, Breast<br>Breast Tumor*<br>Tumor, Breast<br>Tumors, Breast<br>Neoplasms, Breast<br>Breast Cancer<br>Cancer, Breast<br>Mammary Cancer*<br>Cancer, Mammary<br>Cancers, Mammary<br>Malignant Neoplasm of Breast<br>Malignant Tumor of Breast<br>Cancer of Breast<br>Cancer of the Breast<br>Mammary Carcinoma, Human<br>Carcinoma, Human Mammary	Breast cancer

		Carcinomas, Human Mammary<br>Mammary Carcinomas, Human<br>Human Mammary Carcinoma*<br>Mammary Neoplasms, Human<br>Human Mammary Neoplasm*<br>Neoplasm, Human Mammary<br>Neoplasms, Human Mammary<br>Mammary Neoplasm, Human<br>Breast Carcinoma*<br>Carcinoma, Breast<br>Carcinomas, Breast<br>breast gland cancer<br>breast gland neoplasm<br>breast malignan*<br>breast tumor malignant<br>Ca breast OR<br>cancer in the mammary gland<br>cancer of the mammary gland<br>malignancies of the breast<br>malignancy of the breast<br>malignant breast<br>malignant neoplasm of the breast<br>malignant tumor of the breast<br>mamma* cancer<br>mammary gland cancer<br>mammary gland malignan*<br>mammary malignan*	
screening	Early Detection of Cancer	Screening<br>prescreening<br>Detection<br>Mammogra*	screening

Search Strategies:

Instructions: Build the search in PubMed first. Then use Polyglot https://sr-accelerator.com/#/polyglot to translate your search to other relevant databases. **IMPORTANT: You must manually change out the MeSH Terms for the appropriate Emtree terms when translating from PubMed to Embase.**

Filter Applied: 2013 - 2023

Database: PubMed.gov (Includes MEDLINE)

("Early detection of cancer"[Mesh] OR (screening[tiab] OR prescreening[tiab] OR detection[tiab] OR mammogra*[tiab]))

AND

("Breast Neoplasms"[Mesh] OR ("Breast Neoplasm"[tiab] OR "Neoplasm, Breast"[tiab] OR "Breast Tumor*"[tiab] OR "Tumor, Breast"[tiab] OR "Tumors, Breast"[tiab] OR "Neoplasms, Breast"[tiab] OR "Breast Cancer"[tiab] OR "Cancer, Breast"[tiab] OR "Mammary Cancer*"[tiab] OR "Cancer, Mammary"[tiab] OR "Cancers, Mammary"[tiab] OR "Malignant Neoplasm of Breast"[tiab] OR "Malignant Tumor of Breast"[tiab] OR "Cancer of Breast"[tiab] OR "Cancer of the Breast"[tiab] OR "Mammary Carcinoma, Human"[tiab] OR "Human Mammary Carcinoma*"[tiab] OR "Human Mammary Neoplasm*"[tiab] OR "Breast Carcinoma*"[tiab] OR "Carcinoma, Breast"[tiab] OR "Carcinomas, Breast"[tiab] OR "breast malignan*"[tiab] OR "breast tumor malignant "[tiab] OR "Ca breast"[tiab] OR "malignant breast"[tiab] OR "malignant neoplasm of the breast"[tiab] OR "malignant tumor of the breast"[tiab] OR "mamma* cancer"[tiab] OR "mammary gland cancer"[tiab] OR "mammary gland malignan*"[tiab] OR "mammary malignan*"[tiab]))

AND

("Decision support systems, clinical"[Mesh] OR "artificial intelligence"[Mesh] OR "image processing, computer-assisted"[Mesh] OR ("Artificial intelligence"[tiab] OR "Clinical decision aid*"[tiab] OR "Decision aid*"[tiab] OR "Machine learning"[tiab] OR "Learning, machine"[tiab] OR "Intelligence, Artificial"[tiab] OR "Computational Intelligence"[tiab] OR "Intelligence, Computational"[tiab] OR "Machine Intelligence"[tiab] OR "Intelligence, Machine"[tiab] OR "Computer Reasoning"[tiab] OR "Reasoning, Computer"[tiab] OR "Computer Vision System*"[tiab] OR "System, Computer Vision"[tiab] OR "Systems, Computer Vision"[tiab] OR"Clinical Decision Support*"[tiab] OR "Support, Clinical Decision"[tiab] OR "Supports, Clinical Decision"[tiab] OR "Decision Support, Clinical"[tiab]))

Database: Embase.com (Elsevier)

(screening/exp OR (screening:ti,ab OR prescreening:ti,ab OR detection:ti,ab OR mammogra*:ti,ab)) AND ('breat cancer'/exp OR ('breast neoplasm':ti,ab OR 'neoplasm, breast':ti,ab OR 'breast tumor*':ti,ab OR 'tumor, breast':ti,ab OR 'tumors, breast':ti,ab OR 'neoplasms, breast':ti,ab OR 'breast cancer':ti,ab OR 'cancer, breast':ti,ab OR 'mammary cancer*':ti,ab OR 'cancer, mammary':ti,ab OR 'cancers, mammary':ti,ab OR 'malignant neoplasm of breast':ti,ab OR 'malignant tumor of breast':ti,ab OR 'cancer of breast':ti,ab OR 'cancer of the breast':ti,ab OR 'mammary carcinoma, human':ti,ab OR 'human mammary carcinoma*':ti,ab OR 'human mammary neoplasm*':ti,ab OR 'breast carcinoma*':ti,ab OR 'carcinoma, breast':ti,ab OR 'carcinomas, breast':ti,ab OR 'breast malignan*':ti,ab OR 'breast tumor malignant':ti,ab OR 'ca breast':ti,ab OR 'malignant breast':ti,ab OR 'malignant neoplasm of the breast':ti,ab OR 'malignant tumor of the breast':ti,ab OR 'mamma* cancer':ti,ab OR 'mammary gland cancer':ti,ab OR 'mammary gland malignan*':ti,ab OR 'mammary malignan*':ti,ab)) AND ('clinical decision support system'/exp OR 'artificial intelligence'/exp OR 'image processing/exp' OR ('artificial intelligence':ti,ab OR 'clinical decision aid*':ti,ab OR 'decision aid*':ti,ab OR 'machine

learning':ti,ab OR 'learning, machine':ti,ab OR 'intelligence, artificial':ti,ab OR 'computational intelligence':ti,ab OR 'intelligence, computational':ti,ab OR 'machine intelligence':ti,ab OR 'intelligence, machine':ti,ab OR 'computer reasoning':ti,ab OR 'reasoning, computer':ti,ab OR 'computer vision system*':ti,ab OR 'system, computer vision':ti,ab OR 'systems, computer vision':ti,ab OR 'clinical decision support*':ti,ab OR 'support, clinical decision':ti,ab OR 'supports, clinical decision':ti,ab OR 'decision support, clinical':ti,ab))

Database: Cochrane Library

(screening:ti,ab OR prescreening:ti,ab OR detection:ti,ab OR mammogra*:ti,ab)

AND

(“Breast Neoplasm”:ti,ab OR “Neoplasm, Breast”:ti,ab OR “Breast Tumor*”:ti,ab OR “Tumor, Breast”:ti,ab OR “Tumors, Breast”:ti,ab OR “Neoplasms, Breast”:ti,ab OR “Breast Cancer”:ti,ab OR “Cancer, Breast”:ti,ab OR “Mammary Cancer*”:ti,ab OR “Cancer, Mammary”:ti,ab OR “Cancers, Mammary”:ti,ab OR “Malignant Neoplasm of Breast”:ti,ab OR “Malignant Tumor of Breast”:ti,ab OR “Cancer of Breast”:ti,ab OR “Cancer of the Breast”:ti,ab OR “Mammary Carcinoma, Human”:ti,ab OR “Human Mammary Carcinoma*”:ti,ab OR “Human Mammary Neoplasm*”:ti,ab OR “Breast Carcinoma*”:ti,ab OR “Carcinoma, Breast”:ti,ab OR “Carcinomas, Breast”:ti,ab OR “breast malignan*”:ti,ab OR “breast tumor malignant”:ti,ab OR “Ca breast”:ti,ab OR “malignant breast”:ti,ab OR “malignant neoplasm of the breast”:ti,ab OR “malignant tumor of the breast”:ti,ab OR “mamma* cancer”:ti,ab OR “mammary gland cancer”:ti,ab OR “mammary gland malignan*”:ti,ab OR “mammary malignan*”:ti,ab)

AND

(“Artificial intelligence”:ti,ab OR “Clinical decision aid*”:ti,ab OR “Decision aid*”:ti,ab OR “Machine learning”:ti,ab OR “Learning, machine”:ti,ab OR “Intelligence, Artificial”:ti,ab OR “Computational Intelligence”:ti,ab OR “Intelligence, Computational”:ti,ab OR “Machine Intelligence”:ti,ab OR “Intelligence, Machine”:ti,ab OR “Computer Reasoning”:ti,ab OR “Reasoning, Computer”:ti,ab OR “Computer Vision System*”:ti,ab OR “System, Computer Vision”:ti,ab OR “Systems, Computer Vision”:ti,ab OR “Clinical Decision Support*”:ti,ab OR “Support, Clinical Decision”:ti,ab OR “Supports, Clinical Decision”:ti,ab OR “Decision Support, Clinical”:ti,ab)

Database: Web of Science

((TI=screening OR AB=screening) OR (TI=prescreening OR AB=prescreening) OR (TI=detection OR AB=detection) OR (TI=mammogra* OR AB=mammogra*))

AND

((TI="Breast Neoplasm" OR AB="Breast Neoplasm") OR (TI="Neoplasm,

Breast" OR AB="Neoplasm, Breast") OR (TI="Breast Tumor*" OR AB="Breast Tumor*") OR (TI="Tumor, Breast" OR AB="Tumor, Breast") OR (TI="Tumors, Breast" OR AB="Tumors, Breast") OR (TI="Neoplasms, Breast" OR AB="Neoplasms, Breast") OR (TI="Breast Cancer" OR AB="Breast Cancer") OR (TI="Cancer, Breast" OR AB="Cancer, Breast") OR (TI="Mammary Cancer*" OR AB="Mammary Cancer*") OR (TI="Cancer, Mammary" OR AB="Cancer, Mammary") OR (TI="Cancers, Mammary" OR AB="Cancers, Mammary") OR (TI="Malignant Neoplasm of Breast" OR AB="Malignant Neoplasm of Breast") OR (TI="Malignant Tumor of Breast" OR AB="Malignant Tumor of Breast") OR (TI="Cancer of Breast" OR AB="Cancer of Breast") OR (TI="Cancer of the Breast" OR AB="Cancer of the Breast") OR (TI="Mammary Carcinoma, Human" OR AB="Mammary Carcinoma, Human") OR (TI="Human Mammary Carcinoma*" OR AB="Human Mammary Carcinoma*") OR (TI="Human Mammary Neoplasm*" OR AB="Human Mammary Neoplasm*") OR (TI="Breast Carcinoma*" OR AB="Breast Carcinoma*") OR (TI="Carcinoma, Breast" OR AB="Carcinoma, Breast") OR (TI="Carcinomas, Breast" OR AB="Carcinomas, Breast") OR (TI="breast malignan*" OR AB="breast malignan*") OR (TI="breast tumor malignant" OR AB="breast tumor malignant") OR (TI="Ca breast" OR AB="Ca breast") OR (TI="malignant breast" OR AB="malignant breast") OR (TI="malignant neoplasm of the breast" OR AB="malignant neoplasm of the breast") OR (TI="malignant tumor of the breast" OR AB="malignant tumor of the breast") OR (TI="mamma* cancer" OR AB="mamma* cancer") OR (TI="mammary gland cancer" OR AB="mammary gland cancer") OR (TI="mammary gland malignan*" OR AB="mammary gland malignan*") OR (TI="mammary malignan*" OR AB="mammary malignan*"))
AND
((TI="Artificial intelligence" OR AB="Artificial intelligence") OR (TI="Clinical decision aid*" OR AB="Clinical decision aid*") OR (TI="Decision aid*" OR AB="Decision aid*") OR (TI="Machine learning" OR AB="Machine learning") OR (TI="Learning, machine" OR AB="Learning, machine") OR (TI="Intelligence, Artificial" OR AB="Intelligence, Artificial") OR (TI="Computational Intelligence" OR AB="Computational Intelligence") OR (TI="Intelligence, Computational" OR AB="Intelligence, Computational") OR (TI="Machine Intelligence" OR AB="Machine Intelligence") OR (TI="Intelligence, Machine" OR AB="Intelligence, Machine") OR (TI="Computer Reasoning" OR AB="Computer Reasoning") OR (TI="Reasoning, Computer" OR AB="Reasoning, Computer") OR (TI="Computer Vision System*" OR AB="Computer Vision System*") OR (TI="System, Computer Vision" OR AB="System, Computer Vision") OR (TI="Systems, Computer Vision" OR AB="Systems, Computer Vision") OR (TI="Clinical Decision Support*" OR AB="Clinical Decision Support*") OR (TI="Support, Clinical Decision" OR AB="Support, Clinical Decision") OR (TI="Supports, Clinical Decision" OR AB="Supports, Clinical Decision") OR (TI="Decision Support, Clinical" OR AB="Decision Support, Clinical"))

Date Searches Were Exported:

Instructions: Export the results from each database as an RIS file or an NBIB file for PubMed. Then upload those files to a review software tool like https://www.rayyan.ai/ Make a note of the date you export the searches for the methods section of your paper.

9/29/2023

PubMed = 3,054

Embase = 1,455

Cochrane = 60

Web of Science = 1,245
